# Supplementary material for: Active site specificity profiling datasets of matrix metalloproteinases (MMPs) 1, 2, 3, 7, 8, 9, 12, 13 and 14
Source: Data Brief. 2016 Feb 22;7:299–310. doi: 10.1016/j.dib.2016.02.036 (PMC4777984; doi:10.1016/j.dib.2016.02.036)
Supplement: Supplementary file 10 — Supplementary material [file mmc10.zip › WebPICS_hMMP9_T_1%/subsite_coop.html]

 

PICS results


- # About
- # Manual
- # Analysis
- # Results

- Redraw
- Seqlogo
- Dependency
- Coop

**Potential subsite cooperativity analysis for hMMP9\_T\_1%**   
Minimum difference for subsite dependency was set to +- 10 percentage-points  
Subsite dependency was checked for all positional occurences above 1 x natural abundance  
**Subsite dependency check is restricted to P3 - P3'**

|  |  |  |  |
| --- | --- | --- | --- |
| Fixed residue | Affected residue(s) | Change (percentage-points) | Vice-Versa change |
| P3\_A | P1\_N | 15.9 | 20.6 |
| P3\_A | P1\_S | 13.0 | 12.0 |
| P3\_A | P1prime\_M | 11.1 | 23.9 |
| P3\_A | P2prime\_L | 19.9 | 17.3 |
| P3\_C | P2prime\_F | 45.7 | 15.3 |
| P3\_V | P1\_D | 16.6 | 23.9 |
| P3\_V | P1prime\_M | 18.8 | 40.6 |
| P3\_V | P2prime\_H | 15.9 | 20.6 |
| P2\_C | P2prime\_C | 46.4 | 18.6 |
| P2\_F | P1\_P | 24.6 | 12.4 |
| P2\_F | P1prime\_Q | 26.8 | 17.9 |
| P2\_F | P1prime\_V | 41.3 | 20.7 |
| P2\_F | P2prime\_I | 39.1 | 15.7 |
| P2\_F | P3prime\_H | 12.4 | 12.4 |
| P2\_F | P3prime\_N | 28.2 | 24.3 |
| P2\_G | P1\_E | 11.0 | 12.0 |
| P2\_G | P1\_Q | 11.7 | 14.2 |
| P2\_H | P1\_W | 14.5 | 29.0 |
| P2\_H | P1prime\_Q | 26.8 | 17.9 |
| P2\_H | P2prime\_F | 12.4 | 12.4 |
| P2\_H | P3prime\_H | 12.4 | 12.4 |
| P2\_M | P1prime\_V | 24.6 | 18.5 |
| P2\_M | P2prime\_M | 27.5 | 31.0 |
| P2\_M | P3prime\_N | 17.1 | 22.1 |
| P2\_Q | P1\_N | 17.8 | 14.2 |
| P2\_Q | P2prime\_V | 25.9 | 13.0 |
| P2\_S | P1\_S | 12.1 | 15.6 |
| P2\_V | P1\_D | 23.5 | 26.1 |
| P2\_V | P1prime\_C | 16.4 | 32.8 |
| P2\_V | P2prime\_M | 14.2 | 17.8 |
| P2\_Y | P1\_H | 11.4 | 19.9 |
| P2\_Y | P1\_N | 21.4 | 14.9 |
| P2\_Y | P1\_S | 32.8 | 16.3 |
| P2\_Y | P1prime\_C | 10.7 | 14.9 |
| P1\_A | P3prime\_A | 13.2 | 12.0 |
| P1\_C | P1prime\_M | 29.0 | 14.5 |
| P1\_D | P2prime\_M | 16.4 | 18.5 |
| P1\_E | P3prime\_S | 23.3 | 10.2 |
| P1\_G | P1prime\_I | -14.3 | -12.1 |
| P1\_H | P2prime\_Q | 39.9 | 11.4 |
| P1\_H | P3prime\_H | 20.7 | 13.8 |
| P1\_N | P1prime\_M | 15.7 | 26.1 |
| P1\_N | P2prime\_V | 28.4 | 17.8 |
| P1\_P | P1prime\_Q | 10.2 | 13.5 |
| P1\_P | P2prime\_T | 10.9 | 16.3 |
| P1\_P | P3prime\_H | 12.4 | 24.6 |
| P1\_Q | P2prime\_I | 22.4 | 13.5 |
| P1\_S | P2prime\_H | 14.2 | 19.9 |
| P1\_W | P1prime\_W | 31.1 | 31.1 |
| P1\_W | P2prime\_T | 27.5 | 10.3 |
| P1\_W | P3prime\_H | 29.0 | 14.5 |
| P1prime\_I | P2prime\_Q | 13.0 | 24.1 |
| P1prime\_M | P2prime\_H | 26.1 | 15.7 |
| P1prime\_Q | P2prime\_C | 18.6 | 33.5 |
| P1prime\_Q | P2prime\_Q | 23.2 | 14.9 |
| P1prime\_Q | P3prime\_H | 17.9 | 26.8 |
| P1prime\_V | P2prime\_I | 14.1 | 11.3 |
| P1prime\_V | P2prime\_M | 10.9 | 16.3 |
| P1prime\_V | P3prime\_N | 11.6 | 19.9 |
| P1prime\_W | P2prime\_M | 27.5 | 10.3 |
| P1prime\_W | P2prime\_T | 27.5 | 10.3 |
| P1prime\_W | P3prime\_H | 29.0 | 14.5 |
| P2prime\_I | P3prime\_N | 14.9 | 32.0 |
| P2prime\_L | P3prime\_G | 34.6 | 14.8 |
| P2prime\_T | P3prime\_H | 20.7 | 27.5 |

  
**PICS analysis of protease: hMMP9\_T\_1%**  
138 cleavage sites analyzed  
PICS library made with (T)rypsin, (G)luC or (C)hymotrypsin:   
Cutoff for graphic display: 2 x natural abundance  

|  |  |
| --- | --- |
| Positional occurences  (table for total and relative (in %) values) | Occurences relative to natural abundance (table) |
|  |  |
